# Screening of Microalgae for Bioactivity with Antiviral, Antibacterial, Anti-Inflammatory and Anti-Cancer Assays

**DOI:** 10.3390/biology13040255

**Published:** 2024-04-12

**Authors:** Jorge Hernández-Urcera, Alejandro Romero, Pedro Cruz, Vitor Vasconcelos, Antonio Figueras, Beatriz Novoa, Francisco Rodríguez

**Affiliations:** 1 Centro Oceanográfico de Vigo (IEO, CSIC), 36390 Vigo, Spain; jurcera@iim.csic.es; 2 Instituto de Investigaciones Marinas (IIM, CSIC), 36208 Vigo, Spain; aromero@iim.csic.es (A.R.); antoniofigueras@iim.csic.es (A.F.); 3 Centro Interdisciplinar de Investigação Marinha e Ambiental (CIIMAR), 4450-208 Matosinhos, Portugal; pedrocruz95@gmail.com (P.C.); vmvascon@fc.up.pt (V.V.); 4 Faculdade de Ciências, Universidade do Porto, 4169-007 Porto, Portugal

**Keywords:** microalgae, bioactive compounds, zebrafish, inflammation, antiviral activity, antibacterial activity, anti-cancer activity

## Abstract

**Simple Summary:**

Numerous biotechnological applications exist that have been obtained from marine organisms. Nonetheless, despite their diversity and potential for massive growth, marine microalgae have been less studied than other groups. The aim of the present study was to add new information about microalgae to the existing research that shows the bioactivity of marine microalgae in diverse assays conducted by our research group. First, we selected representative species from different algal groups available in our laboratory and then their biomass was harvested to perform several bioactivity tests. Several strains yielded positive results for antibacterial, antiviral and anti-inflammatory activities. These findings act as an essential indicator for future work targeting the structural characterization of the compounds responsible and their potential application in biomedicine.

**Abstract:**

Marine microalgae are a rich reservoir of natural compounds, including bioactives. Nonetheless, these organisms remain fairly unexplored despite their potential biotechnological applications. Culture collections with diverse taxonomic groups and lifestyles are a good source to unlock this potential and discover new molecules for multiple applications such as the treatment of human pathologies or the production of aquaculture species. In the present work extracts from thirty-three strains (including twenty dinoflagellates, four diatoms and nine strains from seven other algal classes), cultivated under identical conditions, were examined for their antiviral, antibacterial, anti-inflammatory and anti-cancer activities. Among these, antiviral and anti-inflammatory activities were detected in a few strains while the antibacterial tests showed positive results in most assays. In turn, most trials did not show any anti-cancer activity. Significant differences were observed between species within the same class, in particular dinoflagellates, which were better represented in this study. These preliminary findings pave the way for an in-depth characterization of the extracts with highest signals in each test, the identification of the compounds responsible for the biological activities found and a further screening of the CCVIEO culture collection.

## 1. Introduction

Marine organisms are an important source of proteins and provide a broad range of health-promoting bioactive compounds with multiple applications in diverse biotechnological and pharmacological sectors such as human health, nutraceuticals, cosmetics, well-being and even animal production [1,2]. Microorganisms included under the umbrella of phytoplankton such as cyanobacteria and microalgae are underexploited as a source of bioactive compounds [3,4,5]. Those microorganisms are of great interest since they are highly taxonomically diverse, showing a complex evolutionary history (as compared, e.g., to land plants). Their ecophysiological diversity confers a high metabolic plasticity, allowing them to respond and adapt to a changing environment by producing bioactive secondary metabolites (reviewed in Saide et al. [6]). Marine planktonic cyanobacteria and microalgae are able to synthetize a large number of potential bioactive compounds including pigments, lipids, glycolipids, alkaloids, terpenoids, ribosomal and non-ribosomal peptides, polyketides, phenolic acids, vitamins, flavonoids and macrolides, among others [4,6,7,8,9], but in many cases the active principles are yet unknown [10,11]. A significant advantage of using microalgae lies in their relatively easy growth and the fact that they can build up a high level of biomass in a short generation time. The ability to produce bioactive compounds is species-specific and depends on several factors such as the environmental parameters (growth medium, temperature, light intensity, pH), length of the growth phase and treatment of the biomass before the extraction [10,12,13,14,15]. Moreover, in some cases the production and release of active metabolites occurs when microalgae are grown in mixed crops or under specific mixotrophic conditions [16,17].

Pharmacological and biological activities have been reported for almost all secondary metabolites produced by microalgae. Those properties included, among others, antibacterial, antiviral, antifungal and antialgal activities [18,19,20,21,22], antioxidant [23,24], anti-inflammatory and immunomodulatory activities [9,10,25,26,27,28] and anti-proliferative and anticancer activity [12,29,30,31]. Moreover, compounds derived from microalgae are useful for the prevention and treatment of other human pathologies such as diabetes, hypertension, artherosclerosis and osteoporosis [6]. Microalgae-derived products have also been applied to the aquaculture industry [32,33]. These organisms are included as food supplements because of their nutritional values and their immunomodulatory properties to mitigate stressful conditions in crustacean and fish production [34,35].

Bacterial and viral diseases are one of the major aquaculture challenges responsible, in some cases, for high mortalities and significant economic losses. Aquaculture fish are affected by several groups of pathogenic viruses such as the birnavirus (IPNV), orthomyxovirus (ISAV), rhabdovirus (SVCV, IHNV and VHSV), adenovirus and herpesvirus (reviewed in Kim and Leong [36]). In particular, the spring viremia of carp virus (SVCV) is responsible for elevated mortalities in carp cultures worldwide [37] and the Office of International Epizootic (OIE) must be notified when it is detected [38]. Although it causes substantial economic losses to the aquaculture industry, there are no effective therapies for its prevention and treatment [39,40]. The major bacterial diseases in aquaculture animals include *Aeromonas* sp. and *Pseudomonas* sp. in many freshwater species, or *Vibrio* sp. in marine ones [41,42,43]. In particular, *Aeromonas hydrophyla* infection affects different freshwater fish species such as common carps, goldfish, eel, catfish and tilapia [44] and is also described as an emergent pathogen for the cultured freshwater shrimp *Litopenaeus vannamei* [45]. Other opportunistic bacteria such as *Micrococcus luteus* can also induce lethal infections when the animal is immunologically compromised [46]. In this context, bioactive compounds from microalgae emerge as a potential source of antiviral components [47,48,49] and are also a plausible alternative to antibiotics to treat bacterial infections [10,50,51,52].

Microalgae also have the ability to induce immunomodulation in fish and confer protection against bacterial infections [35,53,54,55]. The identification and isolation of compounds with anti-inflammatory activity from microalgae are of great interest for human health but also for aquaculture production [56,57,58]. The control of the inflammatory process is relevant since it is involved in many physiological processes such as inflammatory disorders, immune-related diseases and infections [59,60,61]. Microalgae compounds with anti-inflammatory properties include carotenoids, polyunsaturated fatty acids (PUFA) such as eicosapentaenoic and docosahexaenoic acids (EPA and DHA, respectively) and certain sulphated polysaccharides, although other bioactive chemicals are still unknown [10,62].

Available studies on exploring the bioactive compounds in microalgae usually employ diatoms, green algae and dinoflagellates among other groups, but often focus on a limited number of species or bioactivity tests [63]. The screening of a wide range of taxonomic groups and assays is rarely found in the literature [10,13,64]. In that sense, culture collections with different algal classes, life styles and habitats provide biological material of enormous interest to identify new sources of bioactive molecules. These resources enable the samples to undergo different assays under the same conditions, a first step towards finding bioactive fractions and molecules with potential applications in diverse fields, such as biomedicine or aquaculture. 

The present study followed this approach. Taking advantage of the microalgal strains maintained in the CCVIEO culture collection (IEO-CSIC, Spain), a number of phytoplankton groups (nine taxonomic classes) were selected, including toxic dinoflagellate species, and 33 strains were screened for their bioactivity. These tests comprised antiviral, antibacterial, anti-inflammatory and anticancer activities. To our knowledge, many of the organisms included in the present study have not been tested in previous similar works. The obtained results provided some promising findings, concerning antibacterial activity in particular, which deserve to be explored further for their potential applications for human and animal health.

## 2. Results

### 2.1. Antiviral Activity of the Extracts

**Figure 1:**
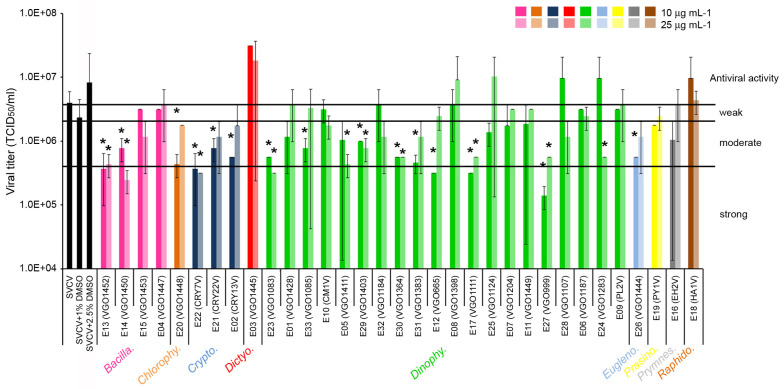
Antiviral activity of the extracts against SVCV. The different algal groups selected are indicated. The viral titer was determined at day 6 post-infection as the viral dilution that causes an infection of 50% of the cell line (TCID_50_). Results were expressed as the mean and SD of four titrations. A *T*-test was used to determine significant differences at *p*-value < 0.05 (*) between control (SVCV infected cells) and cells treated with the extracts at day 6 post-infection. Using the calculated percentage of inhibition (PI), the antiviral activity was scored as strong (PI > 90%), moderate (PI between 50 and 90%) and weak (PI < 50%) according to Monteiro et al. [57].

A total of 17 out of the 33 extracts analyzed showed significant effects against SVCV replication. A significant reduction in the viral titer was obtained at 6 days post infection, although the magnitude of the decrease was always lower than 1 log (Figure 1). The increment in the extract concentration from 10 to 25 μg mL^−1^ did not significantly increase the antiviral activity. Extracts that showed a moderate antiviral activity (PI between 90 and 50%) were obtained from six species of dinoflagellates (genera *Alexandrium* (E33), *Dinophysis* (E5, E29), *Gambierdiscus* (E30, E31), and *Prorocentrum* (E24)) and one diatom of the genus *Nitzschia* (E14). Also, species from other taxonomic classes such as Chlorophyceae (*Tetraselmis*, E20), Cryptophyceae (*Guillardia* E21, and *Teleaulax* E02) and Euglenophyceae (*Eutreptiella*, E26) showed moderate antiviral activity (Figure 1). Finally, extracts from the diatom genus *Chaetoceros* (E13), the dinoflagellates *Alexandrium* (E23), *Gymnodinium* (E12), *Karlodinium* (E17) and *Ostreopsis* (E27) and the cryptophyte *Falcomonas* (E22) showed a strong antiviral activity and significantly reduced the viral titer by up to 90% (PI > 90%) (Figure 1).

### 2.2. Antibacterial Activity of the Extracts

**Figure 2:**
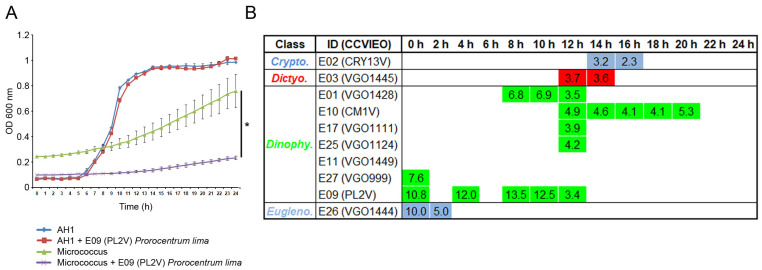
The antibacterial activity of extracts (10 μg mL^−1^) against Gram (−) bacteria. The different algal groups selected are indicated. The bacterial growth was evaluated by measuring the OD600 nm for 24 h; (**A**) The kinetics obtained in samples treated with the extract 9 was selected as representative result. *T*-test was used to determine significant differences at *p*-value < 0.05 (*). (**B**) Tables show the sampling points where the differences in OD600 nm were statistically significant at *p* < 0.05 against *A. hydrophyla*. The percentage of bacterial growth reduction is specified in each sampling point.

The antibacterial activity of the extracts was assayed against both Gram (+) and (−) bacteria using 10 μg mL^−1^ of the extract. The treatment of bacteria with 1% DMSO did not affect the bacterial growth. The evaluation of the OD600 nm during 24 h allowed us to determine the times where the extracts significantly reduced the bacterial growth. The results obtained using extract 9 isolated from the dinoflagellate *Prorocentrum lima* were selected as the representative results (Figure 2A). The growth of the Gram (−) *A. hydrophila* showed logarithmic kinetics and almost reached the highest level after 16 h of incubation. In contrast, an exponential growth was observed in the Gram (+) *M. luteus* (Figure 2A). Only 10 out of 33 extracts significantly reduced the growth of *A. hydrophila* at specific time points and the percentage of reduction was always lower than 13%. The highest antibacterial activity was registered in extracts isolated from the dinoflagellates *A. minutum* (E01) and *P. lima* (E09) after 7 and 8 h of incubation, respectively (Figure 2B).

**Figure 3:**
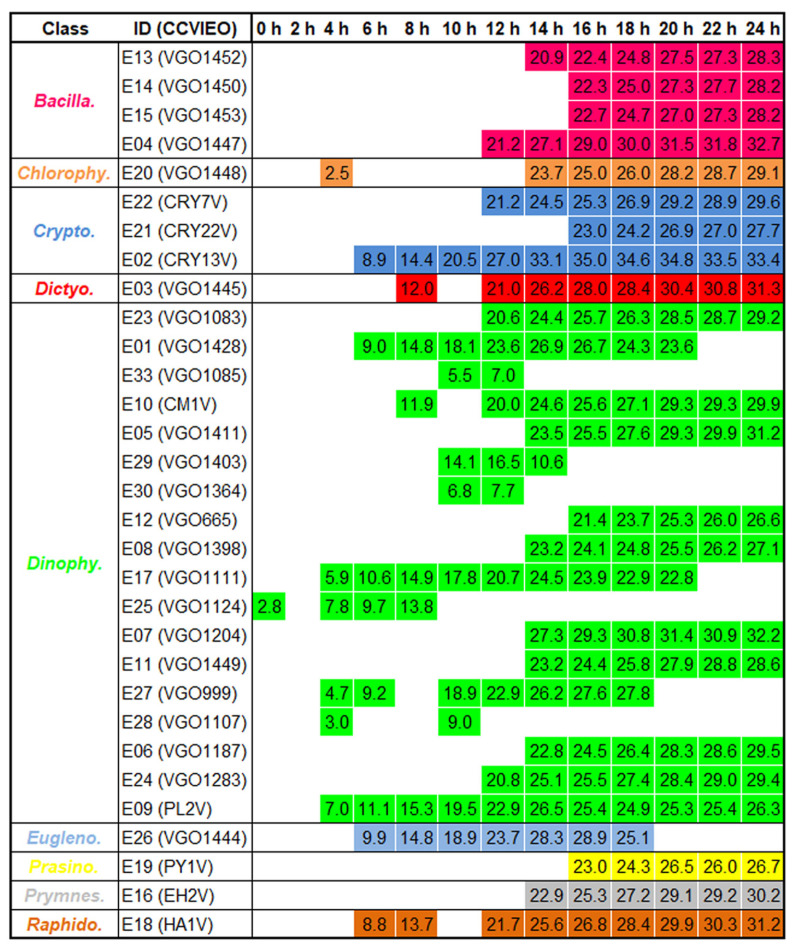
The antibacterial activity of extracts (10 μg mL^−1^) against Gram (+) bacteria. Table shows the sampling points where the differences in OD600 nm were statistically significant at *p* < 0.05 against *M. luteus*. The percentage of bacterial growth reduction is specified in each sampling point. *T*-test was used to determine significant differences at *p*-value < 0.05.

The antibacterial activity against the Gram (+) *M. luteus* was generalized and maintained during all the bacterial incubations (Figure 3). A total of 31 extracts induced significant a reduction in the bacterial growth with an average percentage of reduction close to 30% after 24 h of incubation. The highest percentage of bacterial reduction was registered at 16 h of incubation with the extract isolated from the cryptophyte *Teleaulax amphioxeia* (E02; Figure 3).

### 2.3. Anti-Inflammatory Activity of the Extracts

**Figure 4:**
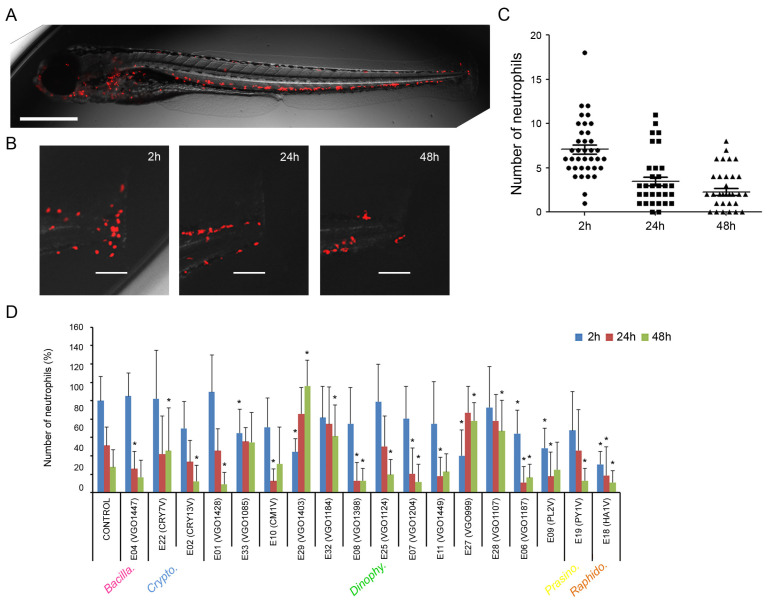
In vivo evaluation of the anti-inflammatory activity of the extracts (at 25 μg mL^−1^) using zebrafish larvae. The different algal groups selected are indicated. (**A**) The transgenic zebrafish larvae Tg(lyz:DsRed2) was used. The lysozyme-expressing cells (neutrophils) are marked in red and can be analyzed in live animals. Scale bar = 500 µm; (**B**) Control animals were injured in the tail and the number of neutrophils was measured at 2 h, 24 h and 48 h. Scale bar = 100 µm; (**C**) Evolution in the number of neutrophils at the injury in control fish. The graph was created using information obtained from 50 animals. (**D**) Effect of the extracts on the number of neutrophils at the injured fin. Results represent the mean and SD. (*) asterisks represent significant differences (*p* < 0.05) compared to controls.

The anti-inflammatory properties of the extracts (at 25 μg mL^−1^) were assayed in vivo using the transgenic zebrafish larvae Tg(lyz:DsRed2). This animal shows the lysozyme-expressing cells (neutrophils) marked in red, allowing for their detection in live animals (Figure 4A). The inflammation was induced by a transversal sectioning of the caudal fin and the number of neutrophils was measured after 2 h, 24 h and 48 h (Figure 4B). The number of neutrophils that migrated to this site increased quickly at 2 h reaching a mean value of seven (±3) cells (Figure 4C). The number of cells significantly decreased in the following sampling points at 24 and 48 h post injury (Figure 4C).

Eighteen out of the thirty-three extracts induced significant changes in the number of neutrophils in any of the sampling points (Figure 4D). At 2 h post injury only six extracts significantly reduced the number of neutrophils. The extract isolated from the raphidophyte *Heterosigma akashiwo* (E18) induced the highest reduction in the number of migrating cells (up to 70%). Moreover, the extracts isolated from *P. lima* (E09), *Ostreopsis siamensis* (E06) and *Dinophysis caudata* (E29) reduced the number of neutrophils by around 60% (Figure 4D). At 24 h, only eight extracts modified the number of cells. The extracts isolated from *O. siamensis* (E06), *P. lima* (E09) and *H. akashiwo* (E18) showed a significant reduction in the number of cells which was maintained from 2 h to 24 h post injury. At this last point (24 h) the extracts reduced the number of cells by around 80% and 60%, respectively (Figure 4D). Interestingly the larvae treated with extracts 27 (*O. fattorussoi*), 28 (*O. ovata*), 29 (*D. caudata*) and 32 (*Gambierdiscus australes*) showed a significant increment in the number of neutrophils at 24 and 48 h. At the end of the experiment (48 h), larvae treated with the extracts isolated from *A. minutum* (E01), *T. amphioxeia* (E02), *O. siamensis* (E06), *Lingulodinium polyedra* (E07), *Heterocapsa minima* (E08), *H. akashiwo* (E18), *Pyramimonas* sp. (E19) and *Kryptoperidinium triquetrum* (E25) showed a significant reduction in the number of cells (Figure 4D).

### 2.4. Cytotoxic Activity of the Extracts in the Cancer Lines

**Figure 5:**
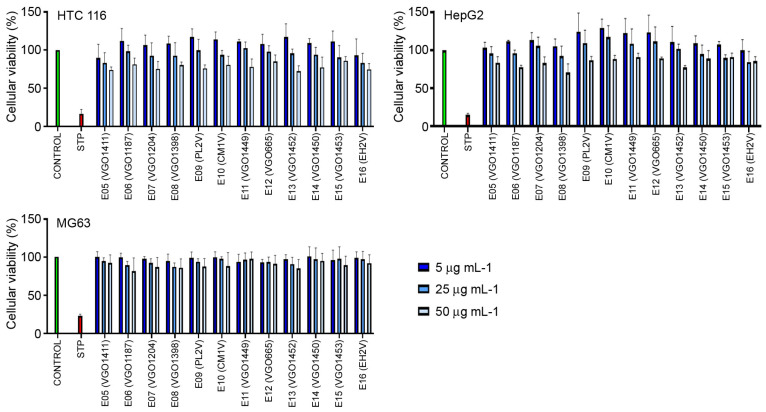
Anti-cancer activity of the extracts against HCT 116, HepG2 and MG-63. Results represent the mean and SD of three independent experiments. STP (staurosporine).

The cytotoxic activity was assayed in vitro using the human cell lines HCT 116, HepG2 and MG-63. None of the extracts showed anti-cancer activity. No significant reduction in the viability of the cells was observed after a 48 h exposure to the extracts at 5, 25 and 50 μg mL^−1^ (Figure 5).

## 3. Discussion

Microalgae represent an interesting source of bioactive compounds [10,13,64] since at least one third of the thirty-three analyzed extracts showed significant effects in some of the bioactivity tests. However, further exploration to identify the responsible fractions and compounds involved in these effects is needed. This work could consider different culture setups, including culture media with certain nutrient limitations, to check if major differences in bioactivity are observed under stressed conditions. Different culture conditions were not examined in the present work and strains were collected at the end of the exponential phase when dense cultures were obtained. However, we cannot provide any clues about the limiting conditions, if any, in our cultures.

### 3.1. Antiviral Activity of the Extracts

The antiviral effect of the extracts is related to the presence of several bioactive molecules such as phenolic and flavonoid components and sulphated polysaccharides [49,57,65,66]. It is suggested that the fatty-acid molecules penetrate the host cell and break down the lipid coat of enveloped viruses, inducing the inactivation of the viruses at their point of entry [67]. The antiviral activity of algae extracts has also been related to the inhibition of the viral RNA polymerase [68]. However, additional studies are required to analyze the mechanisms of action of the algal extracts, focusing on the specific compounds of interest.

In the present study, a total of 17 extracts showed moderate to strong activity against fish SVC virus. This information is in agreement with a similar antiviral activity against fish viruses (IPNV and VHSV) registered for extracts isolated from other marine microalgae species [69]. Our results suggest that the microalgae extracts could persist on the cell surfaces, interfering with the viral replication as was previously proposed by several authors [49,69].

Antiviral activity has been observed in several species of dinoflagellates. The different composition of the extracts between three species of the same genera could explain why the extracts from *Alexandrium mediterraneum* (E23), *A. tamarense* (E33) and *A. minutum* (E01) showed strong, moderate and weak antiviral activity, respectively. The potential antiviral effects of bioactive compounds produced by Alexandrium on marine viruses has already been observed by a reduction in the prevalence of infection with the herpesvirus OSHV-1 µVar in *Crassostrea gigas* when the *A. pacificum* was present [70]. This differential antiviral activity between close species was also observed in *Ostreopsis fattorussoi* (E27) (strong activity) and *O. ovata* (E28) and *O. siamensis* (E06) (no antiviral activity). The differences in the phenolic composition of the extracts or their interaction with other compounds may affect their antiviral activity, as was previously suggested [71]. A strong anti-SVCV activity was also observed in the extract obtained from *Gymnodinium impudicum* (E12). This dinoflagellate has previously been reported to display antiviral activity against several viruses such as the non-enveloped encephalomyocarditis (EMCV) virus that causes death in animal production [19] and against the influenza A virus [72].

Antiviral activity has already been observed in diatoms. Previous studies reported that most species of *Chaetoceros* are rich in fatty acids, such as oleic acid, linoleic acid and alpha-linolenic acid [73]. Although we did not determine the lipid composition of *C. dichatoensis* (E13) their most likely enriched composition in fatty acids could contribute to its strong anti SVCV activity.

Other extracts with antiviral activity belonged to the Cryptophyceae and Chlorophyceae classes. For example, *Falcomonas* sp. (E22) is less known and has recently been explored for bioactive compounds [74]. In contrast, the moderate anti SVCV activity obtained in *T. convolutae* (E20) could be expected since activity against another fish rhabdovirus (VHSV) has already been described in *T. suecica* [75].

### 3.2. Antibacterial Activity

The use of microalgae as sources of natural antibiotics has been extensively explored as an alternative to conventional ones to limit microbial infections in aquaculture. Marine and freshwater microalgae included in different taxonomic groups showed a potent bactericidal activity against both Gram (+) and Gram (−) bacteria [10,50,52]. This activity has been associated with the presence of several metabolites, including sulfated polysaccharides, fatty acids, alkaloids and phenolic compounds [69,76]. For example, extracts containing large amounts of polyunsaturated fatty acids (PUFAs) or saturated fatty acids with 14, 16 or 18 carbons (SFAs) are reported to be more effective against Gram (+) and Gram (−) bacteria, respectively [77]. However, other studies using medicinal plant extracts enriched in SFAs described an antibacterial activity against both bacterial types [78]. In this study, a limited effect against the Gram (−) *A. hydrophyla* and a generalized antibacterial activity against the Gram (+) *M. luteus* were observed. It is necessary to analyze the composition of the extracts to understand the observed results, although there is not a sole compound responsible for the biological activity of the extracts.

The antimicrobial activity of several groups of marine diatoms has been extensively reported [10,20,79,80]. In the present work, the extracts from all the selected diatoms (*C. dichatoensis* (E13), *Nitzschia* sp. (E14), *Pseudo-nitzschia australis* (E15) and *Thalassiosira delicatula* (E04)) presented activity only against the Gram (+) *M. luteus*. This lack of bactericidal effect against Gram (−) bacteria was also described in *Chaetoceros gracilis* [16]. In contrast, other species such as *C. muelleri*, *C. affinis*, *Nitzschia* sp., *P. pseudodelicatissima*, *T. rotula* and *T. weissflogii* showed activity against both bacterial groups [10,79,80,81,82].

Antimicrobial activities of bioactive metabolites, including shellfish toxins of dinoflagellate origins, have also been reported [10,80,83,84]. Only the extracts obtained from the dinoflagellates *A. minutum* (E1) and *P. lima* (E9) showed bactericidal effects against both bacteria (*M. luteus* and *A. hydrophyla*). This result is in line with a strong antibacterial activity of extracts being reported for other dinoflagellates (*P. lima*, *Dinophysis fortii* and *Gambierdiscus* sp.) against several bacteria and fungi (*Aspergillus niger*, *Penicillium funiculosum*, *Candida rugosa*, *Escherichia coli*, *Bacillus megaterium* and *Staphylococcus aureus*) [84,85]. The extracts obtained from *H. minima* (E08) and *Karlodinium veneficum* (E17) also showed a higher activity against the Gram (+) *M. luteus* than the Gram (−) *A. hydrophyla*, as was previously found for *H. circularisquama* and *K. micrum* using other bacterial species [86,87]. Only bactericidal activity against *M. luteus* was observed in the extract isolated from *L. polyedra* (E07), although an extensive activity against both Gram (+) and Gram (−) bacteria has been reported [88].

In this study, antibacterial activity has also been observed in other taxonomic groups such as Chlorophyceae (*T. convolutae*, E20) and Prymnesiophyceae (*Emiliania huxleyi*, E16), in line with previous publications [89,90]. Interestingly, our result suggests an interest in strains from other classes such as Dictyochophyceae, Euglenophyceae, Prasinophyceae and Raphidophyceae as potential reservoirs for antibacterial compounds, as has been suggested for the class Cryptophyceae [74].

### 3.3. Anti-Inflammatory Activity

The anti-inflammatory activity of microalgae extracts has been classically measured in vitro using cell cultures [10,64]. Nevertheless, in the last decade, zebrafish (*Danio rerio*) has emerged as an in vivo model for the screening of anti-inflammatory natural products [91,92]. This in vivo model has proved to be suitable for the investigation of the kinetics of inflammation [93,94,95]. In the present study, a mechanical injury in the fin was performed on zebrafish larvae to attract leukocytes to the damaged area. To our knowledge, this is the first work using this procedure in a zebrafish model to evaluate in vivo the anti-inflammatory activity of several marine microalgae extracts.

Several species of diatoms are known to produce molecules with anti-inflammatory properties [10,11,13,62,81]. In this study only the extract isolated from *T. delicatula* (E04) led to a decrease in the number of neutrophils at 24 h. Although previous information about this species was not available, Asha shalini et al. [81] reported the potent anti-inflammatory activity of a close species (*T. weissflogii*) when assayed in vitro.

The anti-inflammatory activity of dinoflagellates was also described in the literature [10,64], but this activity was previously reported in only two species out of twenty tested in this study (*A. minutum* E01 and *Ostreopsis ovata* E28). Extracts from *A. tamarense* (E33); *D. caudata* (E29); *O. fattorussoi* (E27); *O. siamensis* (E06); and *P. lima* (E09) showed an anti-inflammatory activity as early as 2 h post injury. Those last two extracts (extracts 6 and 9) also maintained their anti-inflammatory state at 24 h post injury. In this context, Lauritano et al. [10] and Asha shalini et al. [81] reported anti-inflammatory activity in closely related dinoflagellate species such as *Coolia malayensis*, *Heterocapsa psammophila*, *Prorocentrum rhathymum* and *P. gracile*. In this study, the anti-inflammatory activity was also observed in extracts from other groups such as the raphidophyte *H. akashiwo* (E18), already described as a beneficial anti-inflammatory agent [92], and the class Cryptophyceae which has recently been targeted in the search for bioactive compounds [74].

### 3.4. Anti-Cancer Activity

Marine microalgae are a reservoir for the discovery of new anti-cancer drugs [12,96,97,98,99]. Preliminary evaluations of the anti-cancer activity of natural extracts are frequently carried out the colorimetric-based MTT/MTS in vitro cell proliferation assay since this is a reliable and economic method to evaluate whole-cell cytotoxicity. However, additional and specific anti-cancer assays can be conducted on positive extracts [100,101]. This methodology has been widely used in the analysis of extracts obtained from several microalgae groups [10,13,64,96,102,103,104,105].

In this work, anti-cancer activity was assessed in vitro on 11 extracts isolated from dinoflagellates and diatoms using different cell lines of human cancers, including colorectal cancer (HCT116), hepatocellular carcinoma (HepG2) and osteosarcoma (MG63). Negative results in all these assays evidenced the lack of any anti-cancer activity, at least under our culture settings and concentrations. This outcome is completely different to previous publications in which anti-cancer activity has been reported in diatoms and dinoflagellates in general [12,97] and particularly in some species closely related to those in our study, such as *Chaetoceros calcitrans* [104], *C. furcellatus* and *C. socialis* [13], *Nitzschia palea* [106], *Pseudo-nitzschia delicatissima*, *Thalassiosira rotula* [97,107], *Coolia malayensis*, *Heterocapsa psammophila*, *Prorocentrum rhathymum* and *P. gracile* [10,64]. In addition to the composition of the extracts with bioactive compounds, it is reported that the anti-cancer activity is dose-dependent and specific for a particular cancer cell line [10]. All those factors could partially explain the discrepancy between the negative results in this work and those from the literature.

## 4. Materials and Methods

### 4.1. The CCVIEO Collection of Harmful Marine Microalgae

The Spanish Institute of Oceanography (IEO-CSIC, Vigo, Spain) maintains a collection of marine microalgae (CCVIEO culture collection) comprising about 250 strains and more than 80 species of microalgae isolated from the Spanish coasts but also from other parts of the world. The collection includes several species associated with harmful algal blooms [108], https://vgohab.com/en/coleccion-de-cultivos/ (accessed on 1 April 2024).

A total of 33 species from 9 different classes were selected in the present study, including strains isolated from the Atlantic coasts (e.g., *Chaetoceros dichatoensis* and *Tetraselmis convolutae*), some of them responsible for harmful algal blooms (e.g., *Alexandrium minutum* and *Dinophysis acuminata*). The distribution of strains in each class was as follows: Bacillariophyceae (4), Chlorophyceae (1), Cryptophyceae (3), Dictyochophyceae (1), Dinophyceae (20), Euglenophyceae (1), Prasinophyceae (1), Prymnesiophyceae (1) and Raphidophyceae (1). The information about species and the strain codes used in the manuscript are specified in Table 1. Strains were identified at species level whenever possible using morphological and molecular data available from previous studies or obtained in the present work, also detailed in Table 1.

### 4.2. Culture and Preparation of Microalgae Extracts

The selected species were grown under specific culture parameters. The cultures were scaled up to a final volume of 1600 mL. The culture parameters were optimized (temperature, light and composition of culture medium) for each species (Table 1). Cultures were grown in three culture chambers kept at 16 °C, 19 °C and 25 °C, under different light intensities depending on the requirements of each strain. A photon irradiance between 80 and 150 μE m^2^s^−1^ of PAR (LED illumination), measured with a QSL-100 irradiometer (Biospherical Instruments Inc., San Diego, CA, USA) and at a 12:12 L:D photoperiod was used (Table 1). The cultures were centrifuged (17,000× *g* for 10 min) to obtain the entire microalgae biomass. The protocol for organic extraction in freeze-dried biomass from the studied strains followed that used for cyanobacteria by Edwards et al. [109].

Some modifications were introduced due to the lower biomass collected in the case of dinoflagellates. The bioactive extracts were obtained from the pellets following a protocol based on H_2_O-methanol and CH_2_Cl_2_-methanol extraction. Briefly, cultures were centrifuged (3000× *g*, 20 min, 4 °C) and the resulting pellets were frozen at −20 °C. Samples were lyophilized for 48 h, and final weights were recorded in each case. Afterwards, 20 mL of methanol was added to the extracts in 50 mL tubes, sonicated in an ultrasonic bath (3 min) and centrifuged again (7500× *g*, 10 min, 4 °C), these steps were repeated three times. Methanol was fully evaporated on a rotary evaporator (Büchi R-200; Flawil, Switzerland). Then, extracts were filtered to eliminate salts using reverse phase columns (Phenomenex, 500 mg mL^−1^, Strata; Torrance, CA, USA). Columns were activated by adding 6 mL of methanol 5%. The non-polar fraction was collected as follows: 6 mL of 100% methanol was added twice and a final rinsing step with 6 mL CH_2_CL_2_ 100% was carried to render a final volume of 18 mL. Then, non-polar extracts were concentrated on a Speedvac (35 °C, 3 h). Dried extracts were weighted again, immediately frozen (−20 °C) and, on the day after, solubilized in DMSO to obtain a stock solution of 1 mg mL^−1^, which was kept at −80 °C until use.

### 4.3. Antiviral and Antibacterial Activity of the Extracts

The antiviral activity of the extracts against the spring viraemia of carp virus (SVCV) was assayed using the ZF4 cell line. The ZF4 cells (ATCC CRL-2050) were cultured at 28 °C in DMEM/F-12, HEPES (Invitrogen, GIBCO) supplemented with 10% fetal bovine serum (FBS Invitrogen, GIBCO, Grandisland, NY, USA), penicillin (100 IU mL^−1^) (Invitrogen, GIBCO) and streptomycin (100 g mL^−1^) (Invitrogen, GIBCO), and buffered with 7.5% sodium bicarbonate (Invitrogen, GIBCO). The SVCV isolate 56/70 was previously propagated on ZF4 cells and titrated in 96-well plates. The effect of the extracts on the viral replication was assayed by mixing the SVC virus with a non-toxic final concentration of 10 and 25 μg mL^−1^ of each extract (1% and 2.5% final concentration of DMSO, respectively). Cells treated with the same concentrations of DMSO and infected were used as positive controls. The plates were incubated at 28 °C for 6 days and examined for cytopathic effects. The virus dilution that causes an infection of 50% of the cell line (TCID_50_) was determined using the Reed–Müench [110] method. Each extract was titrated 4 times. The percentage of inhibition (PI) was calculated to represent the antiviral activity of the extracts using the values of TCID_50_ mL^−1^, as follows: PI = [1 − (T/C)] × 100, with T being the viral titer of treated cells and C the viral titer of the positive control. The antiviral activity was scored as strong (PI > 90%), moderate (PI between 50 and 90%) and weak (PI < 50%) [69].

The antibacterial activity of the different extracts was assayed against both Gram (−) and Gram (+) bacteria. Briefly, the Gram (−) *Aeromonas hydrophyla* AH-1 strain [111] and the Gram (+) *Micrococcus luteus* (aka *lysodeikticus*) were grown on Tryptic Soy Agar (TSA, Scharlab, Barcelona, Spain) plates at 25 °C. Two bacterial suspensions were prepared in Tryptic Soy Broth (TSB, Scharlab, Barcelona, Spain) (at a final concentration of 5 × 10^6^ and 10^6^ CFUs mL^−1^ for *A. hydrophyla* and *M. luteus*, respectively) and mixed with the different extracts to a final concentration of 10 μg mL^−1^ (1% final concentration of DMSO). The bacteria were also treated with 1% DMSO and used as growth control. The bacterial growth was analyzed in a 96-well plate by measuring the OD at 600 nm during 24 h in the GloMax reader (Promega). The percentage of bacterial growth reduction was calculated by using the values obtained in the control wells as 100% of bacterial growth. The experiment was conducted 4 times.

For both assays, the normality of the data was evaluated by a Kolmogorov–Smirnov test and a *T*-test was used to determinate significant differences at *p* > 0.05 by using the GraphPad Prism V7 software.

### 4.4. Anti-Inflammatory Activity of the Extracts

The anti-inflammatory activity of the extracts was assayed in vivo using the transgenic zebrafish larvae Tg(lyz:DsRed2) showing red fluorescent neutrophils [112]. Eggs were obtained by natural spawning and reared at 28 °C. Transection of the caudal fin was performed on three days post-fertilization (3 dpf) larvae using a sapphire single-edge lancet (WPI instrument) at the boundary of the notochord without injury the notochord and the vascular tissue. Animals were previously anesthetized by immersion in water containing 70 mg mL^−1^ tricaine (ethyl 3-aminobenzoate, Sigma-Aldrich). Animals were distributed in a 96-well plate (one animal per well) and immersed in water containing the different extracts at a final concentration of 25 μg mL^−1^. Control animals were treated with water containing the same concentration of DMSO (2.5%). Ten animals were used for each extract. Images of the injured animals were taken at 2 h, 24 h and 48 h using a fluorescent microscope DMi8 (LEICA). The sampling points were selected to analyze the initial cell migration and accumulation (2 h), the resolution of the inflammatory process (24 h) and the late rearrange of neutrophils (48 h) according to the bibliography [93,94,95].

The number of neutrophils in the tail was counted in a 100 µm section anterior to the injury. Multiple focal planes were manually acquired when several neutrophils were overlapped to obtain images at a single-cell resolution. The z-stacks ensured an accurate determination of the number of neutrophils in those thick areas of the tail. The percentage of neutrophils was calculated by considering the number of cells observed in the control animals at 2 h post injury as the 100% of neutrophil migration. All results were expressed as the mean and SD. Significant differences between the data were determined by performing a non-parametric Kruskal–Wallis test with a Dunns post-test using the GraphPad Prism V7 software. A *p*-value of less than 0.05 was considered statistically significant.

### 4.5. Cytotoxic Activity of the Extracts in Cancer Cell Lines

The human colon carcinoma cell line HCT 116 was obtained from Sigma-Aldrich (St. Louis, MS, USA). The human hepatocellular carcinoma and osteosarcoma cell lines, HepG2 and MG-63, respectively, were obtained from the American Culture Collection (ATCC) (Manassas, VA, USA, EUA). The cell lines were maintained in an incubator with an atmosphere of 5% CO_2_ at 37 °C. HCT 116 was cultured in McCoy′s 5A medium (Sigma-Aldrich, St. Louis, MI, USA), while HepG2 and MG-63 were cultured in Dulbecco’s modified Eagle medium (DMEM) (Biowest SAS, Nuaillé, France). Both media were supplemented with 10% fetal bovine serum (FBS) (Biochrom, Berlin, Germany), 1% of penicillin/streptomycin (Biochrom, Berlin, Germany) and 0.1% of amphotericin (GE Healthcare, Little Chafont, Buckinghamshire, UK).

For the assays, the selected extracts were resuspended in dimethyl sulfoxide (DMSO) and the cell lines were seeded at 3.3 × 10^4^ cells mL^−1^ in 96-well plates and incubated for 24 h. After, the cells were exposed to 0.5% of DMSO as a solvent control (maximum solvent concentration used), 1 μM of staurosporine (positive control) and to the concentrations of 5, 25 and 50 μg mL^−1^ of the extracts, for 48 h. 

Cell viability was evaluated using a colorimetric assay with 3-(4,5-Dimethylthiazol-2-yl)-2,5-Diphenyltetrazolium Bromide (MTT reagent). MTT was added to each well, to a final concentration of 200 μg mL^−1^, and incubated for 4 h. The formed formazan crystals were dissolved in 100 μL of DMSO and the absorbance was read in a multi-detection microplate reader (Synergy HT, Biotek, Bart Frederick Shahr, Ebersberg, Germany) at 570 nm. Each cell line was used in three independent assays. For each assay, the values of each treatment were used to calculate the media and then normalized to the solvent control. Then, the data were analyzed using the following equation:

(1) $$Cellviability(\%)=\left(\frac{{Absorbance}_{sample}}{{Absorbance}_{solventcontrol}}\right)\times 100$$

A *T*-test was used to determine significant differences at *p* > 0.05 by using the GraphPad Prism V7 software.

## 5. Conclusions

Microalgae culture collections are a valuable source of bioactive molecules. Promising bioactivities have been registered not only for dinoflagellates and diatoms but also for species belonging to some other groups like cryptophytes and raphidophytes (summarized in Table 2).

Future work will continue this approach towards a more exhaustive exploitation of this CCVIEO culture collection by selecting specific strains, manipulating the culture conditions and identifying the bioactive molecules. Additional efforts should be made to search for new biotechnological and pharmacological applications of these algae in the production of aquaculture species.

## Figures and Tables

**Table 1:** Microalgal strains selected in the present study and culture conditions. L1 codes for L1 medium without silicates.

Class	Species Harmful/Bloom-Forming	ID (CCVIEO) GenBank Acc. Nº	Temp (°C)	Irradiance (μE m^2^s^−1^)	Culture Medium
Bacillariophyceae	*Chaetoceros dichatoensis * −/+	E13 (VGO1452) PP565070	16	150	L1 (+Si)
	*Nitzschia* sp. −/−	E14 (VGO1450) PP565095	16	150	L1 (+Si)
	*Pseudonitzschia australis* +/+	E15 (VGO1453) n.a. *	16	150	L1 (+Si)
	*Thalassiosira delicatula* −/+	E04 (VGO1447) PP565072	16	150	L1 (+Si)
Chlorophyceae	*Tetraselmis convolutae* −/−	E20 (VGO1448) n.a. **	16	150	L1
Cryptophyceae	*Falcomonas* sp. −/−	E22 (CRY7V) PP565085	16	150	L1
	*Guillardia theta* −/−	E21 (CRY22V) PP565107	16	150	L1
	*Teleaulax amphioxeia* −/−	E02 (CRY13V) PP565084	16	150	L1
Dictyochophyceae	*Pseudopedinella elastica* −/−	E03 (VGO1445) PP565071	19	150	L1
Dinophyceae	*Alexandrium mediterraneum* −/+	E23 (VGO1083) n.a.	19	150	L1
	*Alexandrium minutum* +/+	E01 (VGO1428) n.a.	19	150	L1
	*Alexandrium tamarense* −/+	E33 (VGO1085) n.a.	19	150	L1
	*Coolia monotis* −/−	E10 (CM1V) n.a.	19	150	L1
	*Dinophysis acuminata* +/+	E05 (VGO1411) n.a.	19	150	L1 (/20)
	*Dinophysis caudata* +/−	E29 (VGO1403) n.a.	19	150	L1 (/20)
	*Gambierdiscus australes* +/−	E32 (VGO1184) KJ620009	25	80	K/2
	*Gambierdiscus caribaeus* +/−	E30 (VGO1364) MK649659	25	80	K/2
	*Gambierdiscus excentricus* +/−	E31 (VGO1383) MK649640	25	80	K/2
	*Gymnodinium impudicum* −/−	E12 (VGO665) n.a.	19	150	L1
	*Heterocapsa minima* −/+	E08 (VGO1398) PP565093	19	150	L1
	*Karlodinium veneficum* +/+	E17 (VGO1111) PP565094	19	150	L1
	*Kryptoperidinium triquetrum* −/+	E25 (VGO1124) OP256599	19	150	L1
	*Lingulodinium polyedra* +/+	E07 (VGO1204) n.a.	19	150	L1
	*Matsuokaea loeblichii* −/−	E11 (VGO1449) PP565092	19	150	L1
	*Ostreopsis fattorussoi* +/+	E27 (VGO999) KP970827	25	80	L1
	*Ostreopsis* cf. *ovata* +/+	E28 (VGO1107) n.a.	25	80	L1
	*Ostreopsis siamensis* +/+	E06 (VGO1187) KP970826	19	150	L1
	*Prorocentrum hoffmannianum* +/+	E24 (VGO1283) n.a.	25	80	L1
	*Prorocentrum lima* +/+	E09 (PL2V) n.a.	19	150	L1
Euglenophyceae	*Eutreptiella gymnastica* −/+	E26 (VGO1444) n.a.	19	150	L1
Prasinophyceae	*Pyramimonas* sp. −/+	E19 (PY1V) n.a.	19	150	L1
Prymnesiophyceae	*Emiliania huxleyi* −/+	E16 (EH2V) n.a.	19	150	L1
Raphidophyceae	*Heterosigma akashiwo* +/+	E18 (HA1V) AF157385	19	150	L1

All species have been examined by light microscopy (LM) for genus/species identification in previous studies and/or the present one. Genetic sequences not available are indicated by n.a. * Genetic data (rRNA sequencing) was used to identify *P. australis* by FR, but data were lost. ** Directly isolated by FR from its invertebrate host (*Symsagittifera roscoffensis*). Harmful (listed in IOC-UNESCO taxonomic reference list of harmful microalgae: https://www.marinespecies.org/hab; accessed on 1 April 2024) and/or bloom-forming taxa are detailed by “+” or “−” in case of accomplishing or not these criteria.

**Table 2:** Summary of the bioactivities detected in all the microalgae strains. Algal groups are indicated in Table 1. Antiviral activity was scored as strong and moderate (mod). Only significant antibacterial activity was indicated (yes). Eighteen extracts induced significant changes in the number of neutrophils (yes). No cytotoxic activity was registered in the selected extracts. NA: samples not analyzed.

Species	ID	Virus SVCV	G + Bact. *M. luteus*	G − Bact. AH1	Inflammation	Cytotoxicity
*C. dichatoensis*	E13	strong	yes	-	-	-
*Nitzschia* sp.	E14	mod	yes	-	-	-
*P. australis*	E15	-	yes	-	-	-
*T. delicatula*	E04	-	yes	-	yes	NA
*T. convolutae*	E20	mod	yes	-	-	NA
*Falcomonas* sp.	E22	strong	yes	-	yes	NA
*G. theta*	E21	mod	yes	-	-	NA
*T. amphioxeia*	E02	mod	yes	yes	yes	NA
*P. elastica*	E03	-	yes	yes	-	NA
*A. mediterraneum*	E23	strong	yes	-	-	NA
*A. minutum*	E01	-	yes	yes	yes	NA
*A. tamarense*	E33	mod	yes	-	yes	NA
*C. monotis*	E10	-	yes	yes	yes	-
*D. acuminata*	E05	mod	yes	-	-	-
*D. caudata*	E29	mod	yes	-	yes	NA
*G. australes*	E32	-	-	-	yes	NA
*G. caribaeus*	E30	mod	yes	-	-	NA
*G. excentricus*	E31	mod	-	-	-	NA
*G. impudicum*	E12	strong	yes	-	-	-
*H. minima*	E08	-	yes	-	yes	-
*K. veneficum*	E17	strong	yes	yes	-	NA
*K. triquetrum*	E25	-	yes	yes	yes	NA
*L. polyedra*	E07	-	yes	-	yes	-
*M. loeblichii*	E11	-	yes	yes	yes	-
*O. fattorussoi*	E27	strong	yes	yes	yes	NA
*O.* cf. *ovata*	E28	-	yes	--	yes	NA
*O. siamensis*	E06	-	yes	-	yes	-
*P. hoffmannianum*	E24	mod	yes	-	-	NA
*P. lima*	E09	-	yes	yes	yes	-
*E. gymnastica*	E26	mod	yes	yes	-	NA
*Pyramimonas* sp.	E19	-	yes	-	yes	NA
*E. huxleyi*	E16	-	yes	-	-	-
*H. akashiwo*	E18	-	yes	-	yes	NA

## Data Availability

The raw data supporting the conclusions of this article will be made available by the authors on request.
